# Prenatal nitrosatable prescription drug intake, drinking water nitrate, and the risk of stillbirth: a register- and population-based cohort of Danish pregnancies, 1997–2017

**DOI:** 10.1186/s12940-021-00805-z

**Published:** 2021-11-16

**Authors:** Anne Marie Ladehoff Thomsen, Cecilia Høst Ramlau-Hansen, Jörg Schullehner, Ninna Hinchely Ebdrup, Zeyan Liew, Vanessa Coffman, Leslie Stayner, Birgitte Hansen, Jørn Olsen

**Affiliations:** 1 DEFACTUM, Public Health & Health Services Research, Central Denmark Region, Olof Palmes Allé 15, 8200 Aarhus N, Denmark; 2 Department of Public Health, Aarhus University, Aarhus, Denmark; 3 Department of Groundwater and Quaternary Geology Mapping, Geological Survey of Denmark and Greenland, Aarhus, Denmark; 4 Center for Integrated Research-based Research, Aarhus University, Aarhus, Denmark; 5 Department of Environmental Health Sciences, Yale School of Public Health, New Haven, CT USA; 6 Yale Center for Perinatal, Pediatric, and Environmental Epidemiology, Yale School of Public Health, New Haven, USA; 7 Division of Epidemiology and Biostatistics, School of Public Health, University of Illinois at Chicago, Chicago, IL USA; 8 Department of Clinical Epidemiology, Aarhus University Hospital, Aarhus, Denmark

**Keywords:** Nitrosatable drug, Amine, Amide, N-nitroso compounds, Stillbirth, Drinking water nitrate, Cohort study

## Abstract

**Background:**

Nitrosatable drugs commonly prescribed during pregnancy can react with nitrite to form *N*-nitroso compounds which have been associated with an increased risk of stillbirth. Whether maternal residential drinking water nitrate modifies this association is unknown. We investigated, if household drinking water nitrate was associated with stillbirth, and if it modified the association between nitrosatable prescription drug intake and the risk of stillbirth.

**Methods:**

We conducted an individual-level register- and population-based cohort study using 652,810 women with the first recorded singleton pregnancy in the Danish Medical Birth Registry between 1997 and 2017. Nitrosatable drug exposure was recorded by use of the Danish National Patient Registry defined as women with a first redeemed prescription of a nitrosatable drug the first 22 weeks of pregnancy. The reference group was women with no redeemed prescription of a nitrosatable drug in this period. The average individual drinking water nitrate concentration level (mg/L) was calculated in the same period. We categorized nitrosatable drugs as secondary amines, tertiary amines, and amides. Cox hazard regression was used to estimate crude and adjusted hazard ratios with 95% confidence intervals for stillbirth stratified into five categories of nitrate concentrations: ≤1 mg/L, > 1- ≤ 2 mg/L, > 2- ≤ 5 mg/L, > 5- ≤ 25 mg/L, and > 25 mg/L.

**Results:**

Drinking water nitrate exposure in the population was not associated with the risk of stillbirth. Among 100,244 women who had a nitrosatable prescription drug redeemed ≤22 weeks of pregnancy of pregnancy, 418 (0.42%) had a stillbirth compared to 1993 stillbirths (0.36%) among 552,566 referent women. Women with any nitrosatable prescription drug intake and > 1- ≤ 2 mg/L nitrate concentration had an increased risk of stillbirth [adjusted hazard ratio 1.55 (95% confidence interval, 1.15–2.09)] compared with referent women. In the stratified analyses, the highest risk of stillbirth was found among women with secondary amine intake and > 25 mg/L nitrate concentrations [adjusted hazard ratio 3.11 (95% CI, 1.08–8.94)].

**Conclusions:**

The association between nitrosatable prescription drug intake and the risk of stillbirth may depend on the level of nitrate in household drinking water. Evaluations of the effect of nitrosatable drug intake on perinatal outcomes might consider nitrate exposure from drinking water.

**Supplementary Information:**

The online version contains supplementary material available at 10.1186/s12940-021-00805-z.

## Background

An estimated 15–24% of U.S. and Danish women use a nitrosatable drug at some point in time during pregnancy, including common drugs such as antibiotics, asthma medications and antiemetics [1, 2]**.** This broad range of medications contain compounds such as secondary amines, tertiary amines, and amides. Nitrate (NO_3_^**−**^) from drinking water and other nitrate sources is converted to nitrite (NO_2_^**−**^) in the human body and subsequently can react with amines and amides in the gastrointestinal tract to form teratogenic *N*-nitroso compounds (NOCs) under highly acidic environments as in the stomach. NOCs are formed to a greater extent, if the concentration of nitrosating agent is high [3, 4]. NOC formation in the maternal stomach and the subsequent transplacental transmission to the fetus is the most likely source of prenatal exposure to NOCs [5–7]. The teratogenic effect of NOCs may be caused by abnormal development through DNA alkylation [8].

While prenatal nitrosatable drug exposure may cause congenital malformations and preterm birth, few studies have examined the relation between nitrosatable drugs and stillbirth [2, 9–13]. Stillbirth is a rare adverse pregnancy outcome, in Denmark and in other high-income countries, with an estimated rate of 2–5 per 1000 births [14]. The etiology of stillbirth is mainly unknown, but impaired placental function is as a possible major underlying risk factor. Other risk factors include advanced parental age, primiparity, high body mass index (BMI), and maternal smoking during pregnancy, infections, and diseases such as diabetes and hypertension [15–19]. Our results from an earlier study indicated that nitrosatable prescription drug intake (e.g. use of penicillin for infection or terbutaline for asthma) during the first 22 weeks of pregnancy may increase the risk of stillbirth. However, we lacked data on sources of NO3^**−**^ exposure that may modify the association between nitrosatable drug exposure and the risk of stillbirth, including data from drinking water sources [2].

Danish tap water is based completely on groundwater with farming contributing to NO_3_^**−**^ contamination [20]. Roughly 5% of the Danish population is exposed to tap water concentrations > 25 mg/L NO_3_^**−**^, with levels having decreased during the last decades, because of national agricultural nitrogen regulation, local action plans, and infrastructural changes [21]. To protect against infant methemoglobinemia, European Union regulatory limits have been set to 50 mg/L NO_3_^**−**^ in drinking water and a similar level has been set in the U.S. (44 mg/L NO_3_^**−**^). NO_3_^**−**^ exposure below the current regulatory limits has, however, been associated with miscarriage, intrauterine growth restriction, stillbirth, premature birth, and childhood cancer [22–26].

In this study, we examined whether NO_3_^**−**^ in maternal residential drinking water was associated with the risk of stillbirth, and if it modified the association between prenatal nitrosatable prescription drug intake and the risk of stillbirth. Our hypothesis was that the risk of stillbirth would be higher among women who had a nitrosatable prescription drug redeemed during pregnancy and whose residential water NO_3_^**−**^ concentrations were > 25 mg/L compared to those, who did not redeem a nitrosatable prescription drug.

## Methods

### Study design and population

We conducted an individual-level register- and population-based cohort study using data from the Danish Civil Registration System, the Danish Medical Birth Registry, and the Danish National Prescription Register [27–29]. All women with a registered singleton and first recorded pregnancies in Denmark between 1997 and 2017 were identified from the Danish Medical Birth Registry (*n* = 707,467), regardless of their previous pregnancies before the study period. After excluding 54,657 births with coding errors on gestational length, maternal drinking water estimates from private wells, or missing drinking NO3^**−**^ data, our study population included a total of 652,810 mother-child dyads.

### Data sources

The registers utilized in this study offer an exceptional resource with their depth and breadth of data collected. The unique civil personal registration (CPR) number, which has been assigned to nearly all (> 99%) Danish residents since 1968, was used as key identifier to link data between the national registries. In addition, the Danish Civil Registration System holds weekly updated information on date of birth, sex, and residential history [27].

The Danish Medical Birth Registry has information on all births in Denmark from 1973 onwards, including the CPR number of the child, the mother, and the registered father. It also holds information on gestational age based mainly on ultrasound measures and occasionally on last menstrual period, height and weight at birth, birth date, birth outcome (liveborn or death and cause of death), sex, parental age, and number of previous births [28].

The Danish National Prescription Register holds information on redemption of prescribed medication. This register has individual-level data on all prescribed drugs dispensed at any pharmacy in Denmark since 1995 [29]. Pharmacies are required to register all redeemed prescriptions, which are coupled with the reimbursement of expenses from the state. This ensures highly accurate prescription data and completeness has been estimated to be > 97% [30].

### Nitrosatable drug assessment

Detailed procedures for classifying as nitrosatable and for categorizing them based on their functional groups and indications have been discussed in detail elsewhere [1]. We used nitrosatable medicinal compounds lists from published literature to classify nitrosatability, and we included the medication reported to be used between 1997 and 2017 [1, 4, 31]. We included only the medication reported to be used in our population in the study period. All drugs were categorized on the basis of the presence of secondary amine, tertiary amine, and amide functional groups. These categories are not mutually exclusive.

We identified the Anatomical Therapeutic Chemical codes for the categorized drugs and matched these with codes in the Danish National Prescription Register, which also contained information on the date of redemption of the prescription (see Supplementary Table S1 for a complete list of included nitrosatable drugs). All analyses are based on the first nitrosatable prescription drug redeemed from the estimated first day in the last menstrual period (week 0) until the end of week 22 of pregnancy. Women with no redeemed nitrosatable prescription drug during this period were defined as the unexposed comparison.

### Drinking water NO3^−^ assessment

The Danish national geodatabase Jupiter holds drinking water monitoring data [32]. The maternal residential drinking water NO_3_^−^ estimates build on the approach developed and described by Schullehner et al. [33]. In short, annual drinking water production volume weighted average NO_3_^**−**^ concentrations were calculated for public water supply areas and spatio-temporally linked with the geocoded residential history of every person registered in the Danish Civil Registration System. For this study, 107,821 NO_3_^**−**^ samples in 3635 public waterworks between 1993 and 2017 were extracted. NO_3_^**−**^ levels were imputed by interpolation for missing years, if a NO_3_^**−**^ sample was available within 3 years, otherwise the year was assigned as missing NO_3_^**−**^. Individual exposure for each woman was computed as the time-weighted average concentration during pregnancy taking into account changes in residence during pregnancy [34]. The average NO_3_^**−**^ concentration level (mg/L) for each woman was calculated in the period from the estimated first day in the last menstrual period (week 0) until the end of week 22. As private wells are not monitored to the same degree as public supplies, users of private wells (approximately 3% of Danish residents) were excluded from the study population.

### Stillbirth assessment

Before 1 January 2004, stillbirth in Denmark was defined as the birth of a non-vital fetus after ≥28 completed weeks of pregnancy. In 2004 the cut point changed to 22 completed weeks, as in most other countries [35]. To harmonize the study population, we extracted all registered stillbirths born at 22 weeks of pregnancy or later between 1997 and 2017. Pregnancy period was based on the child’s date of birth and gestational age at birth in days.

### Statistical analyses

We investigated, if drinking water NO_3_^−^ in the population was associated with stillbirth by using Cox proportional hazards model with gestational age (days) as the underlying time scale. We estimated crude and adjusted hazard ratios (aHRs) and corresponding 95% confidence intervals (CIs) for stillbirth in relation to five categories of residential drinking water NO_3_^**−**^ concentrations defined a priori according to those previously described cut-off in Coffman et al. 2021 [25]. The first category consists of exposures below the uppermost limit of detection at 1 mg/L. The four other categories were categorized as following: > 1- ≤ 2 mg/L, > 2- ≤ 5 mg/L, > 5- ≤ 25 mg/L and > 25 mg/L.

We also examined whether the association between any nitrosatable prescription drug intake and stillbirth was modified on the multiplicative scale by drinking water NO_3_^**−**^ stratified into the five categories of NO_3_^−^ concentrations. In a secondary analysis, secondary amine, tertiary amine, and amide intake were also stratified into the five categories of NO_3_^**−**^ concentrations to further examine effect medication by drinking water nitrate in these functional groups.

Adjusted analyses included the following covariates identified a priori using existing literature and directed acyclic graphs and obtained from the Danish Medical Birth Registry: parity (continuous), urbanicity of maternal address at birth (five categories), maternal age at delivery (continuous), smoking during pregnancy (yes/no), pregestational BMI (continuous), and highest level of maternal education (six categories) and maternal occupation (seven categories), which were based on the International Standard Class of Occupation and Education codes (ISCO-88 and ISCED) obtained from Statistics Denmark [15, 19, 36]. The proportional hazard assumption and the linearity of covariates were accepted. All analyses were performed using Stata, version 15.0 (StataCorpLP, College Station, TX, USA) and R 4.0 (R Core Team (2021). R: A language and environment for statistical computing. R Foundation for Statistical Computing, Vienna, Austria).

## Results

Our study included 652,810 women with a singleton and first recorded pregnancy leading to birth from 1997 to 2017, regardless of their previous pregnancies before the study period. Among these women, 100,244 (15.3%) had at least one redeemed prescription of a nitrosatable drug during the first 22 weeks of pregnancy. Among those prescribed, the most commonly redeemed drugs were phenoxymethylpenicillin (38.5%), metoclopramide (22.0%), and terbutaline (7.4%) (data not shown). Women, who had a nitrosatable prescription drug intake were generally younger, had a higher BMI, were parous, smoked, were less educated, were more often unemployed, and lived more often in rural and provincial towns compared with referent women, irrespective of NO_3_^**−**^ (Table 1).

**Table 1:** Characteristics of the cohort of women who redeemed, and who did not redeem a nitrosatable prescription drug during the first 22 weeks of pregnancy, 1997–2017, Denmark

Characteristic	Redeemed nitrosatable drug during the first 22 weeks of pregnancy	
	No (*n* = 552,566)	Yes (*n* = 100,244)
Age, y, n (%)		
< 19	5706 (1.0)	1266 (1.3)
19–24	66,476 (12.0)	14,666 (14.6)
24–29	193,577 (35.0)	34,355 (34.3)
29–34	189,329 (34.3)	32,348 (32.3)
> 34	96,972 (17.5)	17,541 (17.5)
Missing	506 (0.0)	68 (0.0)
Body mass index, kg/m^2^, n (%)		
< 18	14,607 (2.6)	2336 (2.3)
18–25	192,003 (34.7)	27,979 (27.9)
25–30	55,658 (10.1)	10,271 (10.2)
> 30	29,124 (5.3)	7361 (7.3)
Missing	261,174 (47.3)	52,297 (52.2)
Parity, n (%)		
Nulliparous	422,921 (76.5)	68,909 (68.7)
Primiparous	76,403 (13.8)	17,930 (17.9)
Multiparous	43,918 (7.9)	11,570 (11.5)
Missing	9324 (1.7)	1835 (1.8)
Smoking during pregnancy, n (%)		
No	401,154 (72.6)	66,726 (66.6)
Yes	82,775 (15.0)	19,259 (19.2)
Missing	68,637 (12.4)	14,259 (14.2)
Maternal level of education, n (%)		
Higher education or PhD	58,747 (10.6)	6820 (6.8)
Middle education	124,762 (22.6)	18,744 (18.7)
Basic education	37,321 (6.8)	5834 (5.8)
High School or vocational	213,658 (38.7)	40,075 (40.0)
Primary school	101,265 (18.3)	26,277 (26.2)
Unknown	13,584 (2.5)	2171 (2.2)
Missing	3229 (0.6)	323 (0.3)
Maternal occupation, n (%)		
Owner of business	10,267 (1.9)	1606 (1.6)
Chief executive or employee with high income	70,443 (12.7)	9270 (9.2)
Employee with middle income	106,673 (19.3)	16,453 (16.4)
Employee with low income	153,572 (27.8)	30,148 (30.1)
Employee unspecified income	81,095 (14.7)	14,403 (14.4)
Pensioners	2301 (0.4)	1087 (1.1)
Unemployed	127,184 (23.0)	27,231 (27.2)
Missing	1031 (0.2)	46 (0.0)
Urbanicity of maternal address at birth, n (%)		
Rural areas^a^	130,413 (23.6)	26,497 (26.4)
Provincial town^b^	143,405 (26.0)	28,956 (28.9)
Provincial city^c^	84,114 (15.2)	13,289 (13.3)
Suburb of capital	63,944 (11.6)	12,403 (12.4)
Capital	121,507 (22.0)	18,205 (18.2)
Missing	9183 (1.7)	894 (0.9)
Household NO_3_^−^ average concentration (mg/L), n (%)		
≤ 1	128,213 (23.2)	23,836 (23.8)
> 1- ≤ 2	170,870 (30.9)	28,624 (28.6)
> 2- ≤ 5	181,868 (32.9)	33,501 (33.4)
> 5- ≤ 25	52,634 (9.5)	10,827 (10.8)
> 25	18,981 (3.4)	3456 (3.4)

^a^ Municipalities in Denmark where the largest town was <10 000 residents

^b^ Municipalities with a town between 10 000-100 000 residents

^c^ Municipalities with town >100 000 residents

We found no association between NO_3_^**−**^ exposure levels in the population and risk of stillbirth in crude and adjusted analyses (Table 2). Among the 100,244 women who had a nitrosatable prescription drug intake during the first 22 weeks of pregnancy, 418 (0.42%) had a recorded stillbirth, while among the 552,566 unexposed women 1993 (0.36%) stillbirths were recorded. Women with any nitrosatable prescription drug intake > 1- ≤ 2 mg/L NO_3_^**−**^ category had a higher risk of stillbirth [aHR 1.55 (95% CI, 1.15–2.09)] compared with referent women. Higher possible risks of stillbirth among women with any nitrosatable prescription drug intake were also estimated in the three higher NO_3_^**−**^ concentration categories compared with referent women, however, the risks were slightly less than those within > 1- ≤ 2 mg/L category (Table 3).

**Table 2:** Hazard ratios (HRs) for stillbirth for given maternal residential drinking water nitrate concentration during the first 22 weeks of pregnancy

Household NO3- average (mg/L)	No stillbirth	Stillbirth	HR (95% CI)	
			Unadjusted	Adjusted^a^
≤1	151,433	616	1	1
> 1- ≤ 2	198,781	713	0.88 (0.79–0.98)	0.97 (0.80–1.18)
> 2- ≤ 5	214,620	749	0.86 (0.78–0.96)	0.88 (0.72–1.07)
> 5- ≤ 25	63,221	240	0.93 (0.80–1.08)	0.95 (0.73–1.24)
> 25	22,344	93	1.01 (0.81–1.26)	1.26 (0.89–1.79)

^a^Adjusted for parity, urbanicity and maternal age, body mass index, smoking, education and occupation

**Table 3:** Hazard ratios (HRs) for stillbirth given any nitrosatable prescription drug intake during the first 22 weeks of pregnancy by levels of maternal drinking water nitrate concentration

Household NO3- average (mg/L)	Any nitrosatable prescription drug intake during the first 22 weeks of pregnancy	No stillbirth	Stillbirth	HR (95% CI)	
				Unadjusted	Adjusted^a^
≤1	No	127,695	518	1	1
	Yes	23,738	98	1.03 (0.83–1.28)	0.89 (0.61–1.30)
> 1- ≤ 2	No	170,289	581	1	1
	Yes	28,492	132	1.38 (1.14–1.67)	1.55 (1.15–2.09)
> 2- ≤ 5	No	181,243	625	1	1
	Yes	33,377	124	1.10 (0.91–1.34)	1.20 (0.87–1.66)
> 5- ≤ 25	No	52,442	192	1	1
	Yes	10,779	48	1.24 (0.91–1.70)	1.32 (0.73–2.37)
> 25	No	18,904	77	1	1
	Yes	3440	16	1.17 (0.68–2.01)	1.32 (0.60–2.90)

^a^Adjusted for parity, urbanicity and maternal age, body mass index, smoking, education and occupation

We found similar results to the main analysis, when we examined secondary amines, tertiary amines, and amides stratified into the five categories of NO_3_^**−**^ concentrations. Overall, the highest risk of stillbirth [aHR 3.11 (95% CI, 1.08–8.94)] was found among women with a secondary amine intake and > 25 mg/L NO_3_^**−**^concentrations compared with referent women. Among women with tertiary amine or amide intake and > 1- ≤ 2 mg/L NO_3_^**−**^ exposure, the risk of stillbirth was higher with an estimated [aHR 1.79 (95% CI, 1.18–2.69)] and an [aHR 1.45 (95% CI, 1.02–2.06)] compared to referent women (Table 4). The associations with each of the most common drugs as exposures (phenoxymethylpenicillin, metoclopramide, terbutaline), stratified into the five categories of NO_3_^**−**^concentrations, were similar to our main analysis (data not shown).

**Table 4:** Hazard ratios (HRs) for stillbirth given prescriptions with secondary amine(s), tertiary amine(s) or amide(s) during the first 22 weeks of pregnancy by levels of maternal household drinking water nitrate concentration

Household NO3- average (mg/L)	Secondary amine intake during the first 22 weeks of pregnancy	No stillbirth	Stillbirth	HR (95% CI)	
				Unadjusted	Adjusted^a^
≤1	No	146,486	594	1	1
	Yes	4947	22	1.12 (0.74–1.72)	1.22 (0.64–2.30)
> 1- ≤ 2	No	192,737	682	1	1
	Yes	6044	31	1.50 (1.05–2.15)	1.39 (0.78–2.49)
> 2- ≤ 5	No	207,954	723	1	1
	Yes	6666	26	1.16 (0.78–1.72)	1.20 (0.65–2.20)
> 5- ≤ 25	No	61,126	234	1	1
	Yes	2095	6	0.78 (0.35–1.76)	0.38 (0.05–2.76)
> 25	No	21,700	87	1	1
	Yes	644	6	2.36 (1.03–5.40)	3.11 (1.08–8.94)
Household NO3- average (mg/L)	Tertiary amine intake during the first 22 weeks of pregnancy	No stillbirth	Stillbirth	HR (95% CI)	
				Unadjusted	Adjusted^a^
≤1	No	141,881	583	1	1
	Yes	9552	33	0.85 (0.60–1.21)	0.55 (0.27–1.12)
> 1- ≤ 2	No	187,809	668	1	1
	Yes	10,972	45	1.18 (0.87–1.59)	1.79 (1.18–2.69)
> 2- ≤ 5	No	201,622	700	1	1
	Yes	12,998	49	1.11 (0.83–1.49)	1.34 (0.85–2.12)
> 5- ≤ 25	No	58,941	221	1	1
	Yes	4280	19	1.21 (0.76–1.94)	1.61 (0.74–3.53)
> 25	No	20,950	87	1	1
	Yes	1394	6	1.07 (0.47–2.44)	1.54 (0.54–4.35)
Household NO3- average (mg/L)	Amide intake during the first 22 weeks of pregnancy	No stillbirth	Stillbirth	HR (95% CI)	
				Unadjusted	Adjusted^a^
≤1	No	134,846	550	1	1
	Yes	16,587	66	0.98 (0.76–1.27)	0.79 (0.50–1.27)
> 1- ≤ 2	No	178,988	622	1	1
	Yes	19,793	91	1.34 (1.08–1.67)	1.45 (1.02–2.06)
> 2- ≤ 5	No	191,278	668	1	1
	Yes	23,342	81	1.01 (0.80–1.27)	1.07 (0.72–1.59)
> 5- ≤ 25	No	55,633	202	1	1
	Yes	7588	38	1.40 (0.99–1.98)	1.50 (0.79–2.86)
> 25	No	19,854	83	1	1
	Yes	2490	10	0.98 (0.51–1.90)	0.90 (0.32–2.54)

^a^Adjusted for parity, urbanicity and maternal age, body mass index, smoking, education and occupation

## Discussion

To the best of our knowledge, this is the first study to examine potential effect measure modification by household NO_3_^−^ tap water on the association between prenatal nitrosatable prescription drug intake and the risk of stillbirth. While drinking water NO_3_^**−**^ concentrations were not associated with the risk of stillbirth, we found that the association between nitrosatable prescription drug intake may be modified by maternal residential drinking water NO_3_^**−**^.

Overall, we observed that the potential effect of any nitrosatable prescription drug intake during the first 22 weeks of pregnancy on the risk of stillbirth was stronger among women within the > 1- ≤ 2 mg/L NO_3_^**−**^ category, whereas comparatively lower, although still higher, risks were observed with higher exposure categories. This may be attributed to survival bias where pregnancy loss or inability to conceive among women in the highest category of exposure may obscure our results. We conditioned our analyses on survival until 22 weeks and the most susceptible fetuses to nitrosatable drug exposure in conjunction with higher levels of NO_3_^**−**^ concentrations may not survive until that gestational age. If survival was influenced by the synergy of the two exposures, the magnitude of our effect may be incorrect, but likely in the correct direction [37]. In stratified analyses, the highest risk of stillbirth was found among women with a secondary amine intake in the > 25 mg/L NO_3_^**−**^ concentration category [aHR 3.11 (95% CI, 1.08–8.94)], however, this finding should be interpreted cautiously due to the low number of cases.

In our previous study, where we found an increased risk of stillbirth with nitrosatable prescription drug intake during the first 22 weeks of pregnancy, we adjusted for municipalities as a proxy measure for drinking water NO_3_^**−**^.^2^ Other birth outcomes studies have focused on nitrosatable drug exposure in conjunction with dietary NO_3_^**−**^ intake and risk of adverse birth outcomes finding that nitrosatable prescription drug intake coupled with dietary NO_2_^**−**^ intake higher than 1.93 and 3.20 mg/day increased the risk of preterm birth and neutral tube defects [38, 39]. Based on what is known about the endogenous formation of NOCs, and our most recent findings, it is important to take drinking water NO_3_^**−**^ into account when assessing the potential harmful effects of nitrosatable drugs on the risk of adverse perinatal outcomes.

There are limitations to our analyses. Information on prescription drug exposure relied entirely on registration of dispensed prescriptions rather than intake of the drugs. However, as patients have to pay a portion of the cost of their redeemed prescriptions, our estimates likely reflect actual drug intake. An additional concern is use of over-the-counter nitrosatable drugs because these are not covered by registry data. However, intake of over-the-counter drugs would result in misclassification of exposed as unexposed and likely bias our findings to the null. We only included drugs according to ATC codes independent of administration route. Topical applied drugs such as nicotine replacement will not lead to formation of NOCs as in the acidic environment in the stomach which would lead to misclassification of unexposed as exposed and likely further bias our findings to the null.

Furthermore, although our classification of nitrosatable drugs is based on the most extensive reviews and up-to-date resources available, there is still the possibility that some nitrosatable drugs have been missed as the components have not been tested for the formation of genotoxic-carcinogenic *N*-nitroso derivatives.

Moreover, nitrosamine impurities have been found in drugs such as Valsartan and Varenicline (https://www.fda.gov/drugs/drug-safety-and-availability/information-about-nitrosamine-impurities-medications, https://www.ema.europa.eu/en/human-regulatory/post-authorisation/referral-procedures/nitrosamine-impurities). However, we did not include these drugs in our exposure, because of the low level of nitrosamines concentrations. These drugs are not classified as nitrosatable drugs, and we doubt that including them in the exposure would bring new insights of importance, and if we did so more drugs should also be considered.

It is expected that exposure misclassification due to consumption of bottled water could also be present. However, we do not consider this to be a large source of bias, because annual bottled water consumption in Denmark is among the lowest in Europe (~ 26 L per person) [40]. We were not able to quantify the amount of water a woman consumed and therefore assumed equivalent consumption for all pregnancies.

Confounding by indication is of concern, as in most non-randomized observational studies. Drugs are prescribed for a medical purpose, and the estimated association could be caused by this underlying condition rather than the drug. We presume however, that if this bias was a major problem, we would have observed variation in associations by drug type according to their functional groups, which we did not. We found similar increased risk for all nitrosatable drug types and the variation in magnitude of the risk between functional groups was small. We did adjust for confounders available from the Danish registries including maternal age, parity, and lifestyle factors; however, we had no information on maternal infections and comorbidities, and therefore residual confounding is possible.

We also did not have information on individual dietary sources of NO_3_^**−**^ and NO_2_^**−**^ (i.e. green vegetables and processed meat), antioxidant supplementation, or vitamin C. Processed red meat products, may also contain low preformed NOCs [41]. However, while high drinking water NO_3_^**−**^ is consistently associated with endogenous capacity, dietary NO_3_^**−**^ is less likely to increase nitrosation due to the presence of nitrosation inhibitors such as polyphenols and vitamin C in vegetables, which are the largest contributor to dietary NO_3_^**−**^intake [42, 43]. Further, dietary factors vary by socioeconomic status and by urbanicity. To partly control for NO_3_^**−**^ intake from dietary sources, we adjusted for maternal education and occupation and urbanicity in the analyses.

Our study had several strengths, including individual-level estimates of NO_3_^**−**^ in maternal drinking water based on measurements performed by certified laboratories. We were able to obtain and link these measurements even as maternal residence changed throughout pregnancy instead of relying on residence at birth. We had a large population and used longitudinal register data, thereby reducing the risk of selection bias and eliminating the risk of recall bias; information on nitrosatable drug exposure was recorded before and independently of the outcome. The classification of stillbirth was based on registry data, with records for inpatient and outpatient treatment in a free of charge antenatal program, which almost all Danish pregnant women take part in [44].

## Conclusions

Drinking water NO^3**−**^ was not associated with risk of stillbirth, however, we found some evidence that the association between prenatal nitrosatable prescription drug intake and the risk of stillbirth may depend on the level of NO^3**−**^ in household drinking water. As nitrosatable prescription drug intake is common during pregnancy and the contamination of groundwater by NO_3_^**−**^ is ubiquitous, it may be important to consider levels of drinking water NO_3_^**−**^, when examining the effect of nitrosatable drug exposure on the risk of perinatal outcomes. Large multicenter studies examining fetal death should be conducted in order to make recommendations to prescribers and regulators.

## Supplementary Information

**Additional file 1 **: **Table S1**. List of included nitrosatable drugs with Anatomical Therapeutic Chemical (ATC) codes.

## Data Availability

The analyses for this study were performed at Statistics Denmark within a strict privacy rules where only researchers who received a signed permit were allowed to do analyses within a secured environment.

## Abbreviations

NO_3_^−^: Nitrate
NO_2_^−^: Nitrite
NOCs: *N*-nitroso compounds
CIs: Confidence intervals (CIs)
HRs: Hazard ratios
aHRs: Adjusted hazard ratios.
