# Supplementary material for: Prenatal nitrosatable prescription drug intake, drinking water nitrate, and the risk of stillbirth: a register- and population-based cohort of Danish pregnancies, 1997–2017
Source: Environ Health. 2021 Nov 16;20:118. doi: 10.1186/s12940-021-00805-z (PMC8594235; doi:10.1186/s12940-021-00805-z)
Supplement: Supplementary file 1 — Additional file 1 : Table S1. List of included nitrosatable drugs with Anatomical Therapeutic Chemical (ATC) codes. [file 12940_2021_805_MOESM1_ESM.docx]

**Table S1:** List of included nitrosatable drugs with Anatomical Therapeutic Chemical (ATC) codes

| Drug | ATC | Compound type^a^ | Drug Class |
| --- | --- | --- | --- |
| Albuterol | R03AC02 | 2 | Asthma, Beta adrenergic |
| Amitriptyline | N06AA09 | 3 | Antidepressant, Tricyclic |
| Amoxicillin | J01CA04 | amide | Anti-infective, Beta lactam |
| Ampicillin | J01CA01 | amide | Anti-infective, Beta lactam |
| Atenolol | C07AB03 | 2, amide | Cardiovascular, Beta blocker |
| Atropine | A03BA01 | 3 | Anticholinergic |
| Caffeine | N06BC01 | 3, amide | Stimulant |
| Carbamazepine | N03AF01 | 3, amide | Antiepileptic |
| Cefalexin | J01DB01 | amide | Anti-infective, Beta lactam |
| Cimetidine | A02BA01 | 2, 3 | Gastrointestinal, H2 blocker |
| Clemastine | R06AA04 | 3 | Antihistamine |
| Clindamycin | J01FF01 | 3, amide | Anti-infective, Macrolide |
| Clonidine | N02CX02 | 2, 3 | Cardiovascular, Antihypertensive |
| Codeine | R05DA04 | 3 | Analgesic, Opiod |
| Dextromethorphan | R05DA09 | 3 | Cough suppressant |
| Diazepam | N05BA01 | 3, amide | Benzodiazepine |
| Diltiazem | C08DB01 | 3, amide | Cardiovascular, Calcium channel blocker |
| Dimenhydrinate | N07CA52 | 3, amide | Antiemetic, Antihistamine |
| Diphenhydramine | R06AA02 | 3 | Antihistamine |
| Dipyrone | N02BB02 | 3 | Analgesic |
| Doxycycline | J01AA02 | 3, amide | Anti-infective, Tetracycline |
| Ephedrine | C01CA26 | 2 | Decongestant |
| Epinephrine(=adrenalin) | C01CA24 | 2 | Asthma |
| Erythromycin | J01FA01 | 3 | Anti-infective, Macrolide |
| Fluoxetine | N06AB03 | 2 | Antidepressant, SSRI |
| Furosemide | C03CA01 | 2, amide | Cardiovascular, diuretic |
| Hydralazine | C02DB02 | 2, 3 | Cardiovascular, Antihypertensive |
| Hydrochlorothiazide | C03EA01 | 2, amide | Cardiovascular, diuretic |
| Hydroxyzine | N05BB01 | 3 | Antihistamine |
| Lorazepam | N05BA06 | amide | Benzodiazepine |
| Metformin | A10BA02 | 2, 3 | Antidiabetic |
| Methadone | N07BC02 | 3 | Analgesic, Opioid |
| Metoclopramide | A03FA01 | 3, amide | Antiemetic |
| Metoprolol | C07AB02 | 2 | Cardiovascular, Beta blocker |
| Metronidazole | J01XD01 | 3 | Anti-infective |
| Morphine | N02AA01 | 3 | Analgesic, Opioid |
| Nicotine | N07BA01 | 3 | Nicotine replacement |
| Nifedipine | C08CA05 | 2 | Cardiovascular, Calcium channel blocker |
| Nortriptyline | N06AA10 | 2 | Antidepressant, Tricyclic |
| Oxycodone | N02AA05 | 3 | Analgesic, Opioid |
| Paroxetine | N06AB05 | 2 | Antidepressant, SSRI |
| Penicillin | J01CE01 | amide | Anti-infective, Beta lactam |
| Phenobarbital | N03AA02 | amide | Antiepileptic |
| Phenoxymethylpenicillin | J01CE02 | amide | Anti-infective, Beta lactam |
| Phenylephrine | C01CA06 | 2 | Decongestant |
| Phenytoin | N03AB05 | amide | Antiepileptic |
| Prochlorperazine | N05AB04 | 3 | Antiemetic |
| Promethazine | R06AD02 | 3 | Antiemetic |
| Propoxyphene | N02AC04 | 3 | Analgesic, Opioid |
| Propranolol | C07AA05 | 2 | Cardiovascular, Beta blocker |
| Pseudoephedrine | R01BA03 | 2 | Decongestant |
| Pyrilamine=Mepyramine | R06AC01 | 3 | Antihistamine |
| Ranitidine | A02BA02 | 2, 3 | Gastrointestinal, H2 blocker |
| Scopolamine | A04AD01 | 3 | Anticholinergic |
| Sulfamethoxazole | J01EE01 | amide | Anti-infective, Sulfonamide |
| Terbutaline | R03AC03/ RO3CC03 | 2 | Asthma, Beta adrenergic |
| Tetracycline | J01AA07 | 3, amide | Anti-infective, Tetracycline |
| Tizanidine | M03BX02 | 2 | Muscle relaxant |
| Verapamil | C08DA01 | 3 | Cardiovascular, Calcium channel blocker |

^a)^ 2: Secondary amine, 3: Tertiary amine
